# Re-Examining Genetic Screening and Oral Contraceptives: A Patient-Centered Review

**DOI:** 10.3390/jpm9010004

**Published:** 2019-01-15

**Authors:** Bridget Hiedemann, Erin Vernon, Bonnie H. Bowie

**Affiliations:** 1 Department of Economics, Seattle University, 901 12th Avenue, Seattle, WA 98122, USA; bgh@seattleu.edu (B.H.); vernoner@seattleu.edu (E.V.); 2 College of Nursing, Seattle University, 901 12th Avenue, Seattle, WA 98122, USA

**Keywords:** thrombophilia screening, genetic screening, combined oral contraceptives, patient-centered care

## Abstract

The World Health Organization classifies combined hormonal contraception as an unacceptable health risk in the presence of a known thrombogenic mutation but advises against routine thrombophilia screening before initiating combined oral contraceptives (COCs) on the grounds of high screening costs and low prevalence. From the perspective of patient-centered care, we examine cost, prevalence, and other published arguments for and against thrombophilia screening before initiating COCs. Our patient-centered review draws on relevant empirical evidence concerning the advantages and disadvantages of thrombophilia screening, while placing the discussion in the broader context of evolving attitudes toward genetic testing and a shifting policy landscape that provides many women direct access to COCs and/or thrombophilia screening. Given variation in prior probabilities of thrombophilia, expected exposure to other risk factors for venous thromboembolism, attitudes towards risk, expected reactions to a positive test result, ability to pay, and concerns about genetic discrimination, we conclude that the current one-size-fits-most approach is not consistent with patient-centered care. Instead, we advocate for greater patient and provider education concerning the implications of thrombophilia screening. Moreover, we recommend offering patients optional thrombophilia screening before initiating COCs.

## 1. Introduction

Over 100 million women worldwide use combined oral contraceptives (COCs) [1]. A highly effective [2] and convenient method of contraception, COCs offer women reproductive autonomy [3]; treatment for premenstrual syndrome, headaches, and acne [1]; and reduced risks of ovarian and endometrial cancer [4].

Using COCs, however, also increases the risks of certain life-threatening conditions including venous thromboembolism (VTE) and/or ischemic stroke, particularly among women with inherited thrombophilia (i.e., thrombogenic mutations) such as the factor V Leiden or the prothrombin gene mutation or deficiencies of protein C, protein S, or antithrombin [4,5]. The World Health Organization (WHO) classifies “a known thrombogenic mutation” as “a condition which represents an unacceptable health risk if [combined hormonal contraception] is used [6].” Conversely, in a clarification for this recommendation, the WHO describes routine thrombophilia screening before initiating COCs as inappropriate in light of the low prevalence of thrombogenic mutations and high screening costs [6]. 

Several studies provide economic evaluations of universal and/or selective thrombophilia screening before initiating COCs from the perspective of a third-party payer [7,8,9,10,11]. Existing studies conclude that universal screening for thrombophilia prior to prescribing COCs is not cost effective [7,8,9]. However, as noted in two independent systematic reviews, most published economic evaluations in this literature lack methodological rigor. For example, most rely on flawed risk estimates and/or lack sensitivity analyses [12,13]. 

Based on a decision tree analysis, one of the highest-quality studies [9] found that universal screening was cost effective when compared to no screening before initiating hormone replacement therapy (HRT) but not before initiating COCs. In the case of both COCs and HRT, however, selective screening (i.e., screening those with a personal or family history of VTE) was more effective than universal screening [9]. 

The existing literature does not include a thorough analysis of the arguments for and against genetic screening from the perspective of the patient. This perspective is particularly relevant in light of an increasing emphasis on patient-centered care, evolving attitudes towards genetic testing, and a changing policy landscape [14]. Policies allowing direct consumer access to thrombophilia testing provide women greater autonomy in this process, as do policies that enable women to access oral contraceptives directly from a pharmacist with or without a prescription [15]. From the perspective of the patient, we analyze published arguments for and against thrombophilia screening before initiating COCs. Our analysis highlights limitations of the current one-size-fits-most approach. We recommend educating patients about the implications of thrombophilia as well as the efficacy and safety of alternative methods of contraception. We also recommend that providers offer patients optional thrombophilia screening before initiating COCs. 

## 2. Background

### 2.1. Patient-Centered Care

Since the Institute of Medicine proposed patient-centered care as one of the six key dimensions of quality in a health care system in 2001 [16], the definition and implementation of this type of care within a clinical setting has been subject to considerable discussion [17,18]. A commonly accepted understanding of this approach to care is one where the patient plays a role in shared decision making and is provided with education to enhance the provider-patient partnership [17,19]. Evidence suggests that patients who most value patient-centered care are female, young, healthy, and well educated [17]. The parallels between those who most value patient-centered care and those considering COCs suggest that many potential COCs users would place a high value on understanding the advantages and disadvantages of thrombophilia screening prior to initiating COCs.

### 2.2. Evolving Attitudes toward Genetic Testing

Along with an increased focus on patient-centered care, there has also been a transition in genetic medicine away from protecting patients from receiving potentially harmful genetic information and toward providing patients with genetic information that could potentially enhance their preventive care [14,20]. For example, Mary-Claire King, recipient of the 2014 Lasker–Koshland Award for her work on breast cancer genetics and human rights, proposes “offer[ing] population screening for unambiguously damaging mutations in these [BRCA1 and BRCA2] genes to all women at about age 30.” She advocates “mov[ing] beyond testing only women in severely affected families to testing women regardless of family history of breast or ovarian cancer—who can then undertake preventive action if they learn they carry a mutation [21].” In the words of King and her colleagues, “women do not benefit from practices that ‘protect’ them from information about their own health. They should have the choice to learn if they carry an actionable mutation in BRCA1 or BRCA2 [22].” Although this viewpoint is not universally accepted within the medical community [23,24], proponents of BRCA screening exemplify a growing acceptance of genetic testing as a preventive measure. 

Another example of changing attitudes involves testing for a gene variant strongly associated with the risk of developing late-onset Alzheimer’s disease, ApoE4. Many providers believe that patients should not find out whether they carry this gene variant, because the disease is currently incurable, not all who have the gene will get Alzheimer’s disease, and there is potential for misunderstanding or mishandling such knowledge [25,26]. However, many members of the ApoE4.Info group (a support group for those testing positive for the gene) have spoken out against this approach stating that this knowledge has empowered them to pursue diet and lifestyle adjustments that may potentially delay onset and engage in activism pushing for further research into more effective treatment [25]. 

Interestingly, recent research has also demonstrated a link between exogenous testosterone treatment and an increased risk for developing a VTE [27,28,29]. In both men and women, the risk for VTE increased when an underlying familial or acquired thrombophilia condition was combined with testosterone therapy. As testosterone therapy for postmenopausal women with low libido becomes more prevalent, more women with undiagnosed thrombophilia may develop VTE and associated complications, including pulmonary emboli [28]. Thus, Glueck et al. [28,29] recommend prescreening patients for thrombophilia prior to commencing testosterone treatment.

This movement toward the use of genetic screening to improve health outcomes of carriers signals a growing general acceptance of genetic testing in situations where positive test results are actionable. Patients who test positive for thrombophilia can use knowledge of their carrier status to reduce their thrombotic risk by selecting other methods of contraception, avoiding hormone replacement therapy, and taking precautions in situations requiring immobilization.

### 2.3. Changing Policy Landscape

In most industries, an individual has easy access to vast amounts of product and service information prior to making important purchasing decisions. Historically, direct access to information about specific services and products has rarely been available to healthcare consumers, but this situation is gradually changing. For example, over the past decade, many countries have approved direct consumer access to certain genetic health risk information including thrombophilia. The company 23andMe provides direct-to-consumer thrombophilia testing along with multiple other genetic health risk tests and ancestry information for around US $200 in several countries including Canada, Denmark, Finland, Ireland, the Netherlands, Sweden, the United Kingdom, and the United States [30]. Patients in these countries can participate in thrombophilia testing without a health care provider’s advice or approval. Unless there is a reversal of the regulation allowing direct-to-consumer access to thrombophilia testing, patients may begin to direct questions about the potential costs and benefits of these tests and/or their results to medical providers. As such, regardless of their opinion on testing, providers will need to be prepared to answer questions about the potential benefits, harms, and implications of such testing for their asymptomatic patients.

In addition to direct access to genetic testing, many patients can access COCs without a provider’s prescription. Currently, in some parts of the US and in much of the world, patients can directly access oral contraceptives without a preliminary assessment by a primary care provider for potential risk factors. Eleven U.S. states (California, Colorado, Hawaii, Idaho, Maryland, New Hampshire, New Mexico, Oregon, Tennessee, Utah, and Washington) and the District of Columbia have passed laws allowing pharmacists to prescribe COCs, while others, such as New York, have pending legislation [15,31]. In some states, women can obtain COCs through online services or smartphone applications [32]. For example, although a physician reviews a patient’s self-reported medical history before authorizing a particular method of birth control, women in 20 states, the District of Columbia, or the Armed Forces in the Americas, Europe, or the Pacific can order COCs through the Nurx app [33]. In contrast to the U.S., most other countries around the world offer over-the-counter oral contraceptives. While some of these countries require some form of medical eligibility assessment, others, including Russia and most South American countries, provide oral contraceptives directly to women with little or no health screening [34]. The intent of the laws is to increase access to birth control by reducing barriers for women who may not have access to a primary care provider. As a consequence of these policies, the dissemination of quality information related to thrombophilia, testing, and COCs no longer rests solely in the hands of health care providers.

## 3. Re-Examining Thrombophilia Screening before Initiating COCs

In light of changing attitudes, technology, product availability, and policies, we revisit the debate concerning thrombophilia screening prior to initiating COCs. In particular, we compiled and examined arguments for and against thrombophilia screening before initiating COCs from the WHO Medical Eligibility Criteria for Contraceptive Use [6] and other published sources. As mentioned earlier, the WHO advises against routine thrombophilia screening before initiating COCs given the high costs of screening and the low prevalence of thrombogenic mutations [6]. Other arguments against routine thrombophilia screening before initiating COCs include the low absolute risk of venous thrombosis [4,35,36,37] even among carriers of thrombogenic mutations using oral contraceptives [4]; concerns that carriers will substitute oral contraceptives with less effective methods of contraception, leading to increased risks of unwanted pregnancy, and, in turn, even greater health risks [4,35,36,38,39]; psychological consequences of both positive and negative test results [36,40]; and the possibility of genetic discrimination [35,36].

At the same time, however, routine thrombophilia screening before initiating COCs affords a “more accurate stratification of the population at risk [38].” Other arguments in favor of routine thrombophilia screening before initiating COCs include the long-term health consequences of VTE [38]; lifetime benefits of awareness of inherited thrombophilia [8,12]; and lost productivity [12].

While existing economic evaluations incorporate some of these costs and benefits, these studies reflect the perspective of a third-party payer. Below we examine each argument from the perspective of the patient. 

### 3.1. Screening Costs

Thrombophilia screening costs vary by genotype(s) and laboratory. For example, health care providers face costs ranging from US $140 to $440 for a factor V Leiden test and US $1500 for a hereditary thrombophilia panel [41]. In some countries, testing for factor V Leiden and the prothrombin gene mutation is available directly to patients for approximately US $200 from 23andme as part of a broader package that includes information about ancestry and health risks. In the United States, patients can currently order factor V Leiden and prothrombin gene testing from Kailos for about US $100 [42].

Even if routine thrombophilia screening before prescribing COCs is not cost effective from the perspective of a third-party payer [7,8,9], an individual patient may be willing to pay out of pocket for screening, depending in part on her attitude toward risk and her ability to pay [43]. For example, more than a third of women interviewed for one study (*n* = 311) expressed willingness to pay for thrombophilia screening out of pocket [43]. Thus, if health care providers do not offer patients thrombophilia screening before initiating COCs, some women may obtain direct-to-consumer genetic health risk screening. Given the concerns about the accuracy of direct-to-consumer genetic testing [44], a positive result on a patient-ordered test may lead to further provider-directed confirmation tests, potentially resulting in increased costs.

### 3.2. Risk of Venous Thromboembolism

One argument against routine thrombophilia screening before initiating COCs concerns the low absolute risk of venous thrombosis [36,37] even among carriers of thrombogenic mutations using oral contraceptives [5]. In its Medical Eligibility Criteria for Contraceptive Use document, the WHO cites research findings where the incidence of deep vein thrombosis among factor V Leiden carriers using COCs has been estimated to be 28.5 per 10,000 women-years [4,6]. A systematic review of the absolute risk of venous thromboembolism among carriers of thrombophilia using COCs suggests this estimate may overstate the risk [45]. On the surface, this argument appears straightforward; however, from a patient’s perspective, there are two important considerations. First, the cumulative risk of thrombosis may be substantial over prolonged COCs use [12]. Thus, among carriers of thrombogenic mutations using COCs for many years, the lifetime risk of thrombosis may not be trivial even in the presence of constant or decreasing annual risks [12]. Second, more than one article points to this absolute risk statistic to note that several women testing positive for thrombophilia could be denied an effective form of birth control even though many would never have experienced VTE [7,46,47]. This reasoning is arguably paternalistic because it removes an opportunity to learn about and weigh a potential health risk. As argued by Caplin and Edelman (2001), the standard of care in a patient-centered environment should allow patients to have shared decision making in situations where basic preventive measures can be taken to avoid morbidity [48]. Even if the absolute risk of VTE among carriers using COCs has been exaggerated in the literature, experts recommend that carriers avoid COCs unless they cannot tolerate other methods of contraception [45]. Patients and providers should also consider that some patients may have an acquired thrombophilia which carries similar risks for VTE and subsequent sequalae [49,50]. For some women, particularly those with the autoimmune disease Lupus, pregnancy or a major illness may trigger an antiphospholipid syndrome and, in turn, thrombophilia and a possible thrombotic event [51]. Whether accessing COCs with or without a prescription, women considering COCs would benefit from a better understanding of potential risk factors as well as short- and long-term risks as many women have little to no knowledge regarding thrombophilia and the risks associated with COCs [43]. As discussed in a later subsection, women who choose not to use COCs on account of thrombophilia have several other highly effective contraceptive options. 

### 3.3. Prevalence of Thrombogenic Mutations and Stratification of the Population at Risk

Another argument against screening concerns the low prevalence of thrombogenic mutations [6]. However, given the high variation in prevalence of thrombophilia by genotype and by ethnicity [52], a one-size-fits-most recommendation concerning routine thrombophilia screening may not be appropriate. For example, while the most common type of inherited thrombophilia, the factor V Leiden mutation, has an overall prevalence of 3 to 8 percent among European and U.S. populations, its worldwide prevalence varies from 10 to 15 percent among whites in Sweden and Greece, among Jordanian Arabs, and in Lebanon, to 0 (or nearly zero) in Japan, Southeast Asia, Africa, and among the indigenous population in Australia [52]. In communities with a relatively high prevalence of thrombophilia, screening affords “a more accurate stratification of the population at risk [38].” In these communities, patients considering COCs would benefit from a more personalized evaluation including a discussion of thrombophilia and its implications. Abstracting from an individual patient’s attitude toward risk, those from ethnic backgrounds with relatively high prevalence may be particularly interested in thrombophilia screening before initiating COCs. Regardless of ethnicity, a patient may appreciate education pertaining to her potential risk factors and related screening options before choosing this method of contraception.

### 3.4. Long-Term Consequences of Venous Thromboembolism and Lost Productivity

While the short-term risk of developing venous thromboembolism is relatively low even among carriers of thrombogenic mutations using COCs, the potential long-term consequences of VTE may be important considerations to a patient. For example, Bennett, Patterson and Noble (2014) found evidence of long-term emotional consequences for patients who developed VTEs. Women, in particular, reported statistically significantly higher trauma and health anxiety scores than men with a mean time elapsed since the VTE event of two years and three months at the time of data collection [53]. VTEs are also associated with diminished psychological well-being and quality of life [54]. Kahn et al. (2005) found that quality of life scores for patients who experienced a VTE were lower than the general population at one month and that, at four months after the event, scores were similar to patients with arthritis or chronic lung disease [54].

In addition to adverse psychological effects, there are also economic ramifications for VTE patients. These include the direct costs of hospitalization and treatment for VTE. Also many VTE patients develop a recurrent VTE within five years following the first event and require anticoagulation for several years, including frequent close monitoring through an anticoagulation clinic [55]. Grosse et al. (2015) estimate costs for treating VTEs over the first year between US $12,000 and $15,000. Long-term complications associated with VTEs include chronic thromboembolic pulmonary hypertension, post-thrombotic syndrome, adverse reactions to anticoagulants, and, as mentioned, reoccurrence of VTEs. Aggregate costs associated with VTE treatment range from US $375,000 to $425,000 per patient [56]. Treatment costs, however, do not include loss of productivity for patients. In their study of 66,005 patients, Brækkan et al. (2016) found that patients with a VTE had a 37% higher risk of a work-related disability than those without a VTE [57]. If the patient had a pulmonary embolism in addition to a deep vein thrombosis, the risk of a work-related disability was 80% [57]. In light of these potential long-term consequences, some patients may prefer to learn their thrombophilia status before initiating COCs. 

### 3.5. Risk of Pregnancy

One frequent argument against screening deserves particular scrutiny—namely, the concern that withholding COCs from women with inherited thrombophilia would lead to a reliance on less effective methods of contraception, leading to a higher risk of pregnancy and, in turn, even greater health risks [4,35,36,38,39,40]. This argument is misleading in light of the availability of highly effective methods of contraception that are safer than COCs for women with inherited thrombophilia [43]. All of the methods listed in Table 1 have the same or lower typical-use failure rates than COCs. For example, the copper-bearing intrauterine device—a method of contraception considered safe for women with a known thrombogenic mutation—has a typical-use failure rate of 0.6–1%, compared to 1–9% for COCs [58,59]. For carriers with a strong preference for oral contraceptives, the progestin-only pill is safer than COCs with a similar typical-use failure rate. When patients testing positive for thrombophilia are counseled about alternative methods of contraception that pose lower health risks without increasing their risk of pregnancy, this rationale for avoiding thrombophilia screening is unwarranted. For example, based on a sample of 2480 women included in a previous study of APC resistance and pregnancy, the 270 women who had tested positive for factor V Leiden did not have statistically or practically significantly higher rates of abortion than the 2210 women who had tested negative even though most carriers who had used COCs discontinued use after learning their test results [60]. 

Moreover, empirical evidence suggests that the use of contraception may be more critical than the specific method of contraception in terms of avoiding unwanted pregnancy. A recent study of contraceptive use and sexual activity among 15–19-year-old women attributes the substantial reduction in adolescent birth rates from 1991 to 2013 to increased use of contraception among adolescents. The authors conclude that “adolescents’ uptake of any method, regardless of its failure rate, markedly reduces this [pregnancy] risk [61].” 

In addition to effectiveness and health risks, cost may influence a patient’s decisions concerning contraception. Thus, withholding COCs from women with inherited thrombophilia might induce women with thrombogenic mutations to substitute less effective methods for COCs if safer alternatives were too expensive. However, costs borne by patients of various methods of contraception are often comparable. In the United States, for example, the Affordable Care Act allows most women with insurance full coverage, without cost sharing, of several contraceptive methods including COCs, progestin-only pills, progestin injections, IUDs with progestin, and copper IUDs [62]. Similarly, many other countries completely or partially subsidize various contraceptive methods [63,64]. Even among women without health insurance, costs of oral contraceptives are not necessarily lower than alternative methods. For example, in the U.S., women without insurance and those with insurance through employers who object to birth control coverage on religious or moral grounds often face higher initial but lower lifetime costs of IUDs than oral contraceptives. While a common copper IUD, ParaGard®, may cost as much as US $875 with a $300 placement fee [2013], the average annual cost of the copper IUD is lower than the annual cost of oral contraceptives (US $240–$600 2013) for women using the IUD for several years [65]. It is worth noting that the relatively higher upfront cost of the IUD can be perceived as a barrier for low income, uninsured patients considering birth control options [66].

In view of the relative effectiveness as well as the relative costs of alternative methods of contraception, the argument that withholding COCs from women with inherited thrombophilia would increase risks of pregnancy and related health risks is both unwarranted and paternalistic. 

### 3.6. Psychological, Clinical, and Behavioral Implications of Thrombophilia Screening

An additional argument against thrombophilia screening before initiating COCs concerns the potential psychological consequences of both positive and negative results. While a positive test result may provoke needless anxiety, a negative test result may provide a false sense of reassurance [36,40,67,68,69]. However, empirical evidence concerning the psychological implications of thrombophilia screening suggests that these concerns are exaggerated. Although a substantial minority of individuals testing positive for APC resistance or the factor V Leiden mutation experienced greater worry following their test results [70], the vast majority of individuals—regardless of carrier status—in several studies reported that they did not regret participating in thrombophilia screening [58,70,71,72]. For example, one study that tested patients for protein C deficiency found that patients perceived the benefits of this knowledge to outweigh the consequences. The authors concluded that “there seem to be few negative psychological consequences of knowing that one is at increased risk for venous thrombosis, except in vulnerable individuals [72].”

As mentioned above, many researchers [40,67,69,73] have expressed concern that a false sense of security among patients with negative test results may cause them to potentially ignore early symptoms of a VTE. As with all genetic information received by a patient, education regarding the interpretation of test results, whether positive or negative, is paramount. In this particular case, patients should be educated that negative thrombophilia test results do not guarantee that a VTE event will never occur. Education on recognizing VTE symptoms should be provided for all, regardless of carrier status. Some have expressed concern that this information is not effectively provided right now [74]. 

In addition, as most types of familial thrombophilia are the result of an autosomal dominant gene, knowledge of this condition could be very informative for first degree relatives of the patient [75]. Knowledge of a first-degree relative’s positive thrombophilia test result could guide prescription decisions and possibly avoid adverse outcomes. Stevens et al. (2016) emphasize that screening should only be performed when the result can be used to influence treatment decisions or patient behavior [75].

Several studies conducted from the perspective of a third-party payer argue that screening is “excessive” because it adds to the already high health care costs without adding much, if any, benefit [73,76]. From the patient’s perspective, the key question concerns whether this knowledge has actionable benefits that could potentially outweigh the costs. Evidence suggests that this may be the case as this knowledge has potential clinical consequences not only regarding COCs use [58] but also pertaining to clinical management of pregnancy, menopause, and surgery [70,77]. Moreover, awareness of thrombophilia [8,77] enables carriers to take precautions in high-risk situations such as long flights or bed rest. Evidence suggests that awareness of thrombophilia motivates some carriers to avoid immobility or long trips [70,71], but the evidence concerning other behavioral implications is mixed [14,70,71]. 

Depending on characteristics of the individual and the results of the test, screening before initiating COCs may have positive or negative psychological and behavioral consequences. However, optional screening enables an individual patient to consider her possible reactions to the test results. Would she prefer “knowing for the sake of doing [69]”—in this case avoiding COCs and mitigating other risks in the presence of a positive test result—or selecting a contraceptive method without knowing whether she has thrombophilia?

### 3.7. Risk of Genetic Discrimination

From the patient’s perspective, the potential for genetic discrimination in employment and/or insurance may be a compelling reason to avoid thrombophilia screening. Policies such as the United States Genetic Information Nondiscrimination Act of 2008 (GINA), the Charter of Fundamental Rights of the European Union, the United Kingdom’s Concordat and Moratorium on Genetics and Insurance, and Australia’s 2008 Amendment to the Disability Discrimination Act [78] prohibit genetic discrimination in employment and/or health insurance. However, there are still situations where awareness of genetic health risk factors could be costly for an individual. For example, while GINA prohibits discrimination on the basis of genetic information in employment and health insurance, the law does not cover life, disability or long-term care insurance [79]. Research suggests that even asymptotic thrombophilia patients face challenges obtaining life or disability insurance and/or higher premiums [80]. Concern also exists among patients that current legislation may not provide enough protection against genetic discrimination even in the areas of employment and health insurance [79]. Given the potential barriers to life and/or disability insurance, some patients may prefer not to participate in thrombophilia screening.

## 4. Discussion

Given variation in women’s willingness to pay out of pocket for thrombophilia screening, prevalence of thrombophilia by ethnicity, and other relevant characteristics, a one-size-fits-most approach to thrombophilia screening before initiating COCs is not consistent with patient-centered care. While the relatively high prevalence of factor V Leiden in certain parts of Europe and the Middle East may make testing before initiating COCs worthwhile for many women [52], most African and Asian women would probably not find testing worthwhile given the negligible to low prevalence of this gene variant in Asian and African populations [52]. In addition, some patients may have other contraindications for COCs that would negate the value of screening as a decision aid for COCs use. Such contraindications include current or past diagnoses of certain types of cancer, smoking past the age of 35, and migraines with aura [6]. In addition, factors such as obesity and family history of VTEs also increase the risk of VTEs during COCs use which, although categorized by the WHO as category 2 risks meaning they are conditions “where the advantages of using the method generally outweigh the theoretical or proven risks [6]”, may cause risk averse patients to choose other methods of birth control in any case.

Moreover, evidence suggests that concerns about adverse psychological effects of thrombophilia testing are exaggerated for most women [72]. Awareness of the implications of thrombophilia and the option to participate in screening before initiating COCs enable an individual patient to weigh the benefits of actionable knowledge against the costs of potential anxiety. In light of the availability of other effective methods of contraception, concern about denying asymptomatic carriers an effective method of contraception is unwarranted and paternalistic. The accumulation of risks over prolonged COCs use, long-term consequences of VTE, and clinical relevance of awareness of thrombophilia are serious considerations that may persuade an informed patient to participate in thrombophilia screening, while the risk of genetic discrimination may dissuade an informed patient from participating.

Since the cost–benefit analysis varies by patient, we recommend greater patient education concerning thrombophilia, the potential implications of a positive test result, the limitations of a negative test result, and the efficacy as well as the risks of alternative methods of contraception. We concur with previous experts who have advocated for offering women an informed choice concerning thrombophilia screening before initiating COCs [36,48,74]. Not only is our recommendation consistent with that of Caplin and Edelman [48] and of Legnani et al. [36] in the specific context of thrombophilia screening before initiating COCs, but it also echoes King et al.’s [22] arguments for offering women BRCA1 and BRCA2 screening as part of routine care. Optional thrombophilia screening under the direction of a healthcare provider reduces the potential harm of thrombophilia screening while offering the patient the opportunity to make a more informed decision and preserving the patient’s autonomy in this process. 

The limitations of our recommendation are worth acknowledging. Offering women an informed choice concerning thrombophilia screening before initiating COCs poses challenges for healthcare providers and patients. Educating patients about thrombophilia and issues to consider before deciding whether to participate in screening may increase the duration of provider/patient contraception discussions. However, high-quality educational materials available to patients in clinics, pharmacies, and on websites and apps could mitigate these costs. Greater participation in thrombophilia screening increases the need for providers to understand the implications of thrombophilia not only for contraceptive contraindications, but also other high-risk situations. Patients who participate in thrombophilia screening before initiating COCs need to realize that anyone—regardless of thrombophilia status—could develop a VTE. 

As discussed by Burke and Trinidad (2016), testing without proper education may cause more harm than good. They argue that in some cases, direct-to-consumer marketing and subsequent test results may result in unrealistic patient expectations of what their physicians can do with the findings which, in turn, could cause unnecessary confusion, distress, and patient mistrust of physicians [81]. In addition, direct-to-consumer testing may result in false positives that could cost the patient further confirmation tests and unnecessary worry. Specifically, Tandy-Connor et al. (2018) found that 40% of gene variants identified through direct-to-consumer testing were not confirmed when rigorous confirmation techniques were undertaken [44]. In light of valid concerns about the lack of patient education and counseling regarding direct-to-consumer genetic testing as well as the rate of false positives, it is important that health care providers offer thrombophilia screening before initiating COCs to minimize the potential for misunderstandings and errors.

Our recommendation also poses political challenges given historical and current controversies concerning contraception and reproductive rights. The ability to delay childbirth increases the likelihood that women will stay in school, thereby enhancing the economic and health status of their families [82]. Thus, politicians and healthcare professionals have worked to make oral contraceptives more readily available, particularly to women who face barriers to obtaining birth control. With the notable exception of the 1995 pill scare provoked by the United Kingdom’s Committee on Safety Medicines’ warning linking COCs with increased VTE risk [83], healthcare providers have tended to emphasize the benefits of the pill such as reproductive autonomy, efficacy, and convenience. According to health activist and writer Holly Grigg-Spall, concerns about provoking another “pill scare” and divisiveness concerning reproductive rights inhibit frank discussions about the potential health consequences of COCs [84]. She argues that many view open discussion about the pill’s side effects as a “betrayal of the feminist cause [84].” 

Another political hurdle concerns inequitable access to healthcare [14,85]. As scholars have acknowledged in the context of pregnancy management, thrombophilia screening at a cost that is easily affordable for some while out of reach for others “could further exacerbate healthcare disparities between different socioeconomic groups [85].” Thus, by drawing attention to the potential side effects of the pill and potentially contributing to a two-tiered medical system, offering optional thrombophilia screening before initiating COCs could be interpreted by some critics as potentially anti-feminist and inequitable. Nevertheless, we conclude that more patient-centered, higher-quality care could be achieved by empowering women with greater awareness of thrombophilia and the option to obtain thrombophilia screening before initiating COCs.

## 5. Conclusions

Notwithstanding concerns about direct-to-consumer genetic testing in general and arguments against thrombophilia screening before initiating COCs in particular, many women have the autonomy and the means to access direct-to-consumer thrombophilia testing regardless of practice guidelines. Thus, from a clinical perspective, our research highlights the need for appropriate strategies for providing efficient and effective education for patients considering COCs as well as more extensive guidelines regarding counseling and treating patients with direct-to-consumer genetic test results. More broadly, our research highlights the increasing importance of incorporating the patient’s perspective in clinical and economic evaluations of genetic testing.

## Figures and Tables

**Table 1:** Safety of highly effective contraceptive methods for women with thrombophilia.

Contraceptive Method	Eligibility for Women with Known Thrombogenic Mutations [6]	Typical-Use Failure Rate [58,59]
Combined Oral Contraceptives	Unacceptable health risk	1–9%
Progestin-Only Pill	Advantages of using the method generally outweigh the theoretical or proven risks	1–9%
Progestin Implant		0.05–1%
Progestin Injections		1–6%
Levenorgestrel IUD		0.2–1%
Copper-Bearing IUD	No restriction	0.6–1%

IUD = intrauterine device.
